# Supplementary figures and images for: Multimodal Brain Signal Complexity Predicts Human Intelligence
Source: eNeuro. 2023 Feb 2;10(2):ENEURO.0345-22.2022. doi: 10.1523/ENEURO.0345-22.2022 (PMC9910576; doi:10.1523/ENEURO.0345-22.2022)

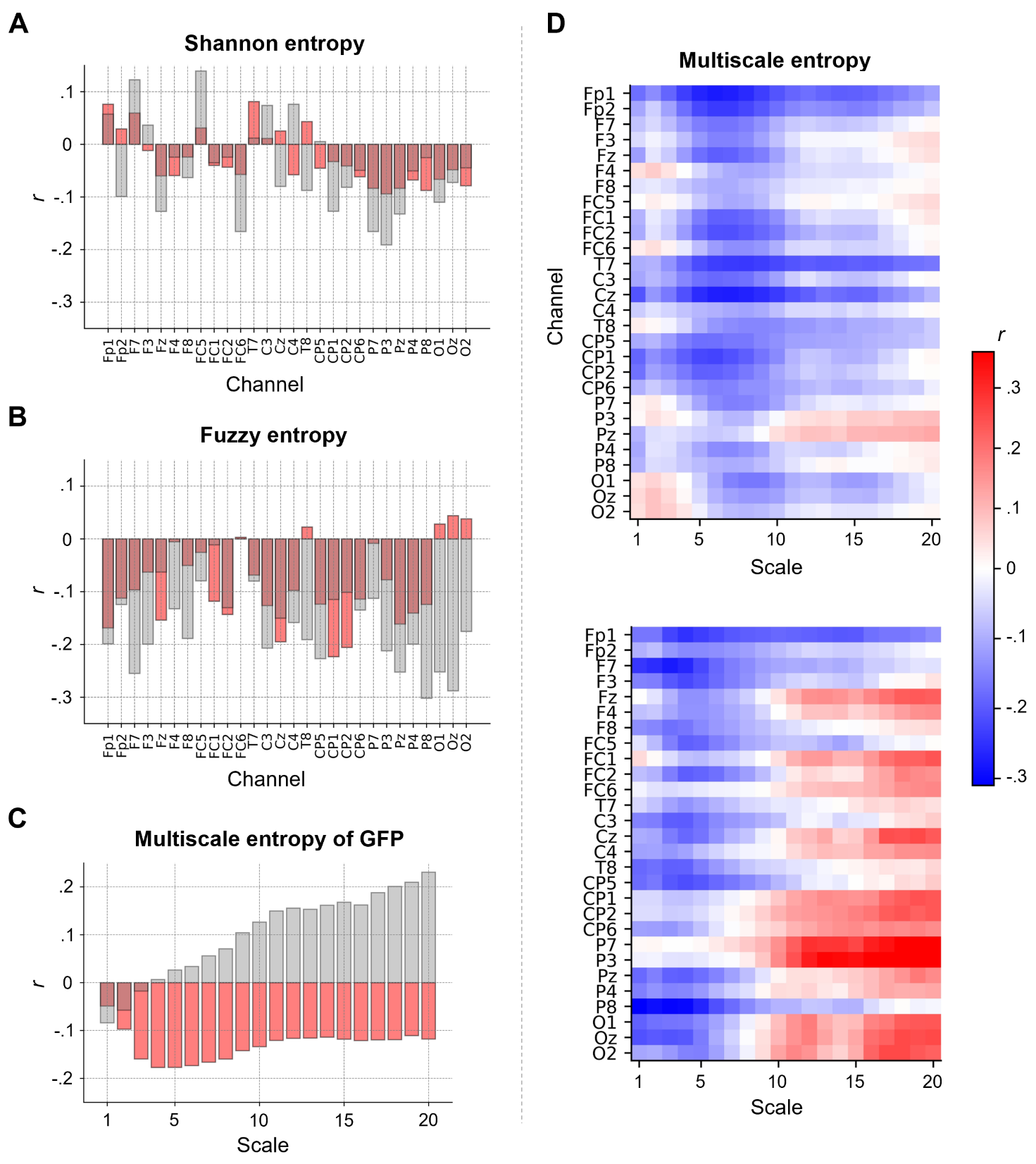

Supplement: Extended Data Figure 6-1 — The association between intelligence and intrinsic brain signal entropy depends on electroencephalography (EEG) channel, time scale and study sample. Pearson correlations r (not controlled for age, sex, and number of removed epochs) between intelligence (RAPM; Raven and Court, 1998) and (A) Shannon entropy for each EEG channel, (B) Fuzzy entropy for each EEG channel, and (C) multiscale entropy (MSE), indexing the sample entropy at different coarse-grained time series (temporal scales), of the global field power (GFP) at time scales 1 to 20. Associations found in the main sample (N = 144) are depicted in red, associations found in the replication sample (N = 57) are illustrated in gray. D, Pearson correlations r (not controlled for age, sex, and number of removed epochs) between intelligence and MSE computed for the time scales 1 to 20 and each EEG channel. Upper panel, Main sample. Lower panel, Replication sample. Figure Contributions: Jonas A. Thiele and Kirsten Hilger designed research. Jonas A. Thiele, Aylin Richter, and Kirsten Hilger performed research. Jonas A. Thiele and Aylin Richter analyzed data. Download Figure 6-1, TIF file. [file enu-eN-NWR-0345-22-s03.tif]
